# Experimental and Numerical Studies of Tool Wear Processes in the Nibbling Process

**DOI:** 10.3390/ma15010107

**Published:** 2021-12-24

**Authors:** Łukasz Bohdal, Leon Kukiełka, Radosław Patyk, Katarzyna Kośka, Jarosław Chodór, Konrad Czyżewski

**Affiliations:** Department of Mechanical Engineering, Koszalin University of Technology, Racławicka 15-17 Street, 75-620 Koszalin, Poland; leon.kukielka@tu.koszalin.pl (L.K.); radoslaw.patyk@tu.koszalin.pl (R.P.); katarzyna.koska@s.tu.koszalin.pl (K.K.); jaroslaw.chodor@tu.koszalin.pl (J.C.); konrad15kcz@gmail.com (K.C.)

**Keywords:** nibbling, punch wear, punch surface analysis, workpiece cut surface analysis, FEM analysis, fatigue wear resistance

## Abstract

The work concerns an analysis of the wear mechanisms of punches in the nibbling process. The nibbling process is the multiple punching of holes or external contours using circular punches, the diameter of which is much smaller than the size of the punched shapes. Analytical, numerical and experimental studies were carried out. In the analytical solution, formulas for determining the pressures in the contact zone were developed, thus enabling a simple estimation of the designed nibbling tools. In numerical studies, the influence of the punch rounding radius on the fatigue wear was investigated. It has been shown that the change in the punch cutting edge radius from r = 0 mm to r = 0.5 mm enables a seven-fold increase in the fatigue wear resistance. It was found that the change in the punch cutting edge rounding radius has an impact on the quality of the product (the greater the radius r, the worse the technological quality of the product). In experimental studies, the abrasive wear process was primarily investigated. For this purpose, the nibbling process was tested on S235JR + AR steel sheets with tools made of NC11LV/1.2379 steel without any coating and with an AlCrTiN layer. It was found that the special AlCrTiN layer used allowed for an increase in the resistance to abrasive wear, and thus increased the service life by approx. three times. The last element of the work is an assessment of the technological quality of the product after nibbling depending on the degree and type of stamp wear (quantitative and qualitative assessment).

## 1. Introduction

**Figure 1:**
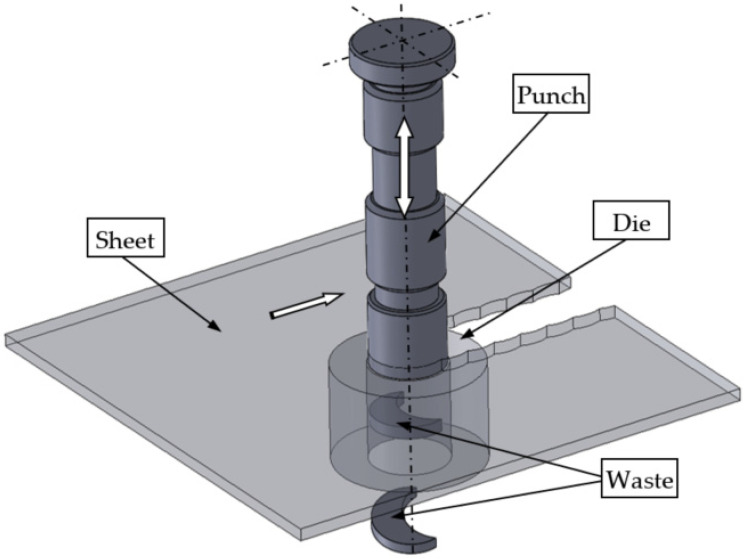
Illustration of the nibbling process.

Nibbling is a multiple punching process. The process is often used in plastic working to produce both simple details and those with complex shapes. This method is a freeform cutting of a sheet metal with a series of cut-outs that, together, create a slot (Figure 1). The sheet is placed on the die with a rear stop position, after which, the punch presses a hole. The punch is then lifted and moved over the plate until the stop position, again positioning the punch that knocks out another hole. The process is repeated while the plate is rotated to create a nonlinear slit [1]. The main challenge in the production lines is to obtain high-quality products with the optimal shape of the cut edge. It should be free of burrs and there should be no concentration of large plastic strains. The quality of the obtained product depends on many process parameters, such as the lubrication method, cutting speed, tool geometry, cutting clearance, punch material properties and punch wear [2,3,4,5].

These parameters are the key factors influencing the physical phenomena occurring in the process, such as the state of material displacement, deformations and visco-plastic stress, as well as its energy consumption. These phenomena affect material separation mechanisms in the cut zone, such as the visco-plastic shear or fracture.

In the literature related to the problems of punching and blanking processes, most publications concern the analysis of the morphology of the cut edge, process optimization and product defects. The latest research also concerns tool wear processes. In industrial practice, the wear of the punch has a significant impact on the production because the critical level of wear causes deviations in the shape of the details [5,6]. Many studies concern analyses related to the correct selection of the technological parameters of punching and blanking in terms of extending the life of the tool [7,8]. Issues related to the mechanism of the formation of burrs on the cut edge, depending on the degree of wear of the punch, are also discussed, and, thanks to which, recommendations are sought to predict their formation and control their height [9,10,11,12]. In order to analyze the shearing mechanisms, wear models implemented in the numerical formulations are used [13,14].

The analysis of the state of knowledge shows that the service life of tools is significantly influenced by working conditions, the mechanical and physical properties of the sheet and the tool design, as well as advanced production processes that are applied on the tool [11,15,16,17].

Increasing the durability of the blanking tools can be achieved by using materials with a higher hardness, such as high speed steel (HSS) or cemented carbides. One of the development directions is applying wear-resistant coatings to the working surfaces of tools. At the moment, research into wear-resistant coatings is continuously developing. The modifications of substrate material, single and/or multi-layered hard coatings and coating technology (PVD, CVD, TD, DLC, etc.) are being investigated [18]. Recently, TiN coatings produced by physical vapor deposition (PVD) have been rapidly finding uses in a wide field of industries, since they possess excellent properties, such as a high wear resistance, low coefficient of friction and good chemical inertness [11,19,20,21]. Some researchers analyzed the influence of AlCrN coatings on the punch wear resistance in blanking [22,23]. It has been shown that the use of these coatings can significantly increase the number of tool cycles. However, the subject has not yet been thoroughly researched.

There are no items on the nibbling process in the literature. In the case of this process, the number of work cycles that are needed to obtain the product is greater than in the case of standard punching or blanking. However, there are lower cutting forces and lower inertia. It is possible to use smaller machine tools. Holes of various diameters can be made without the need to make a new punch and die with different dimensions, which significantly reduces production costs. It is possible to obtain details with a complicated curvilinear outline. Therefore, the recommendations and guidelines for the design of tools are different than in the case of standard punching (especially with regard to the selection of geometrical parameters of the tools and their influence on the tool life).

The literature lacks the results of research and analyses enabling the identification of wear mechanisms and development of guidelines for the design of tool geometry, which will enable the extension of their work cycles. In order to reduce the stress concentration, which can delay crack formation and extend the service life, it is possible to modify the tool geometry by the nibbling process. However, there is no information on how to modify the geometry of the tool so as to not affect the deterioration of the quality of the cut edge, nor information on how to increase the durability of the tools through the use of coatings.

The aim of the study is to assess the impact of the punch coating (AlCrTiN—TIGRAL) on the occurrence and dynamics of the abrasive and adhesive wear process, as well as to analyze and assess the impact of tool design (rounding radii) on the fatigue wear phenomenon (spalling and pitting). Abrasive and adhesive wear is primarily related to the condition of the top layer of tools and the blank. The initiation of this type of wear is random; therefore, its tests should be carried out in a manner that is as close to the real process as possible. Therefore, it was decided that experimental tests would be conducted. The process of form fatigue is more controlled and depends on the pressure distribution in the contact zone; therefore, it is easier to model physically, mathematically and, as a result, computer modeling is easier to perform.

The implementation of the set goal was carried out in two stages: experimental studies of the abrasive and adhesive wear process on a selected example, and numerical studies of the impact of tool geometry on the course of the fatigue wear process (spalling and pitting).

## 2. Materials and Methods

**Figure 2:**
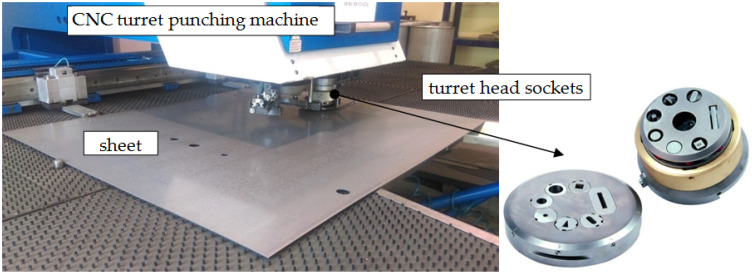
Nibbling equipment.

**Figure 3:**
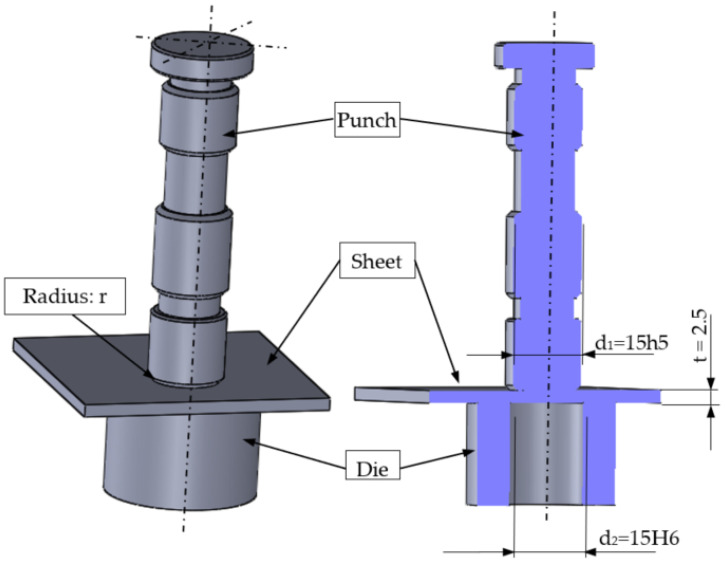
The geometry of nibbling equipment.

The nibbling process was carried out in industrial conditions using CNC turret punching machine BOSCHERT TWIN ROTATION (Boschert GmbH & Co., KG, Lörrach, Germany) (Figure 2). The basic geometry of the punch tool is presented in Figure 3. Tool parameters (height, diameter, number of work cycles of the punch) were saved in the system, and then assigned to specific programs. The punches were mounted in the seven tool turret head sockets. The tool was additionally secured with a safety pin from the inside and a cover. The nibbling process was carried out up to 120,000 cuts. Then, the tools were sharpened. The process speed value was constant (250 cuts/min). The main mechanical properties and chemical compositions of punches made from NC11LV/1.2379 ledeburitic chromium steel are presented in Table 1 and Table 2. The chemical composition presented in Table 2 was developed on the basis of a certificate and confirmed by spectrometric analysis in accordance with the standards PN-EN ISO 9556, PN-EN 24935, PN-EN ISO 10720. All tests were performed at room temperature. The tools were hardened in oil at T = 1020 °C ± 10 °C (control error) and tempered in air at T = 280 °C ± 10 °C.

The workpiece material with a thickness of *t* = 2.5 mm and dimensions of 900 mm × 500 mm made from S235JR + AR structural grade carbon steel was used. The details of properties are given in Table 3 and Table 4. The data presented in Table 3 were averaged on the basis of 10 measurements made on the Zwick/Roell Z400 testing machine (ZwickRoell GmbH & Co., KG, Ulm, Germany) with the use of an extensometer and vision techniques (ARAMIS system, where local elongation was measured). The main AlCrTiN—TIGRAL coat characteristic properties are presented in Table 5 and are manufacturer’s data. The applied coating achieves significantly increased hardness compared to traditional TiN, TiCN and TiAlN coatings. This layer is characterized by its high warm hardness, its oxidation stability and its abrasion resistance. These properties can be attributed to a nano-structure that minimizes crack propagation, especially in case of shear loading within the coating. The cutting clearance used in this study was 16% of the sheet thickness.

Punch coatings were made by using PVD method (physical vapor deposition) by Voestalpine Group using Eifeler technology after proper surface preparation. For the hardness measurements, a Rockwell hardness tester (QATM, Mammelzen, Germany) was used. The samples were prepared according to test standards [22,23], and the KABiD—PRESS testing machine was used in research. Each hardness test was carried out 5 times to obtain a reliable result. In order to analyze the wear resistance, loss of the punches during the process was measured using precision balance METTLER Toledo XS 105 (Mettler Toledo, Warsaw, Poland) with a sensitivity of ±0.0001 g.

Before measurements, the punches were cleaned with acetone and compressed air. The weight loss of each punch was taken three times to assure reliable results. To investigate the influence of punch wear on the hole geometry of the piercing workpiece, Olympus LEXT OLS 4000 Confocal Laser Microscope (Thermo Fisher Scientific, UK) and ATOS Triple Scan system (GOM a ZEISS company, WESTCAM Datentechnik GmbH, Mils, Austria) with professional software for scanning and 3D control was used.

For the measurement of the cut surface quality of the holes, an optical microscope manufactured by Kestler—Vision Engineering Dynascope Ltd. (Vision Engineering, Woking Surrey, UK) with measurement system ND 1300 Quadra-Chek (E-MOTION, Temecula, CA, USA) and a sensitivity of ±0.001 mm was used.

## 3. Results and Discussion

During the process of sheet metal punching, three main types of wear can occur simultaneously: abrasive wear, adhesive wear and fatigue wear, which is both spalling and pitting. Abrasive wear occurs when material loss in the top layer is caused by particle separation due to micro-cutting, scratching or grooving. Such a process occurs when, in the friction areas of the cooperating elements, there are loose or fixed abrasive particles or protruding irregularities of a harder material, which act as localized micro-blades.

Adhesive wear (by tacking the first type) occurs in the micro-areas of the plastic deformation of the surface layer, especially in the highest peaks of roughness. Local metallic tacking of the friction surfaces then arises, followed by the destruction of these joints along with the detachment of metal particles or its smearing on the friction surfaces. Adhesive wear occurs at a sliding friction with low relative velocities and high unit pressures in the areas of the actual contact surface, if the particles of both surfaces are brought closer to the distance of the molecular force. Adhesive wear occurs at the contact of two metallic surfaces, especially when identical metals, characterized by high chemical affinities, are in contact.

Form fatigue wear by peeling (spalling) is a gradual increase in stresses in the surface layer of the associated frictional elements (rolling or skid rolling) at a dry contact within the Hertz stress limits, as a result of the cyclic interaction of contact stresses, where the subsequent formation of microcracks and spreading cause the material particles to fall off of the substrate. It can also occur in insufficiently lubricated components. It manifests itself in local losses of scale-shaped material separated from the ground by friction.

Fatigue wear by pitting is fatigue wear caused by the cyclical impact of contact stresses arising in the surface layers of frictional elements (turning or sliding with a lubricated contact within the limits of Hertz stresses). It is therefore the fatigue wear that occurs in the presence of the lubricant. Due to the multitude of wear processes occurring during sheet metal punching, obtaining a high-quality finished product and high predictable durability of punching tools is a very complex problem.

In the case of dominant abrasive wear, it is recommended to smooth the surface to be punched, to use tools with high hardness coatings and to keep the cutting zone clean.

In the case of predominant adhesive wear, it is recommended to change the design of the tool in such a way as to reduce the contact pressures, smooth the contact surfaces and increase the cutting speed.

In the case of dominant form fatigue wear (both spalling and pitting), the pressures in the contact zone should be reduced, and in the case of spalling, the friction coefficient between the contacting elements (sheet, die and punch) should be reduced. The discussed problem requires the technologist to solve a very complex poly-optimization problem, both in the selection of technological parameters of the punching process (speed, clearance, angles on the tools, rounding radii, type and parameters of lubrication, etc.) and the design of tools.

### 3.1. The Results of the Abrasive Wear Process

#### 3.1.1. Wear Mechanisms Identification

**Figure 4:**
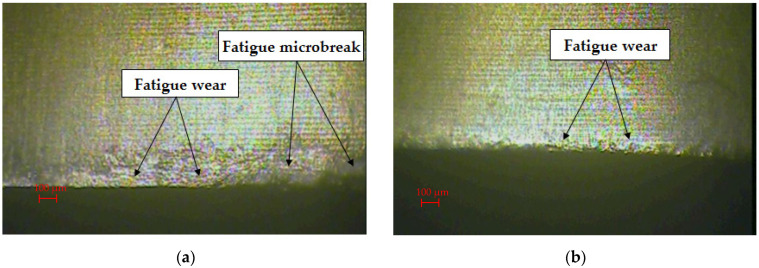
Edge and face wear of the punch at the 70,000 strokes: (**a**) uncoated; (**b**) coated.

**Figure 5:**
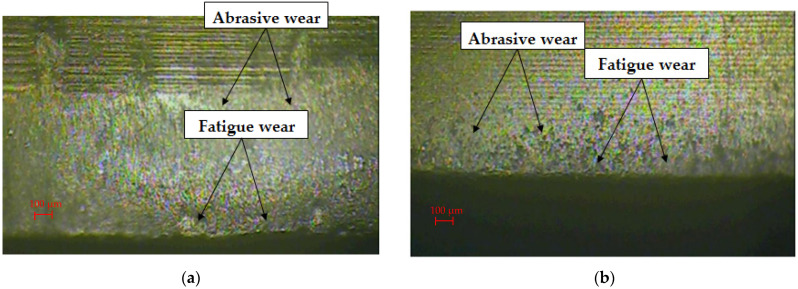
Edge and face wear of the punch at the 120,000 strokes: (**a**) uncoated; (**b**) coated.

In order to identify and analyze the wear mechanisms after the nibbling process, a detailed analysis of the obtained images of the punch’s cutting edges after selected work cycles was performed. Sample images are presented in Figure 4 and Figure 5.

The stamp without coating after 70,000 work cycles had numerous wear signs on the flank surface, such as furrows, scratches and microcracks, which appear to a different degree on the analyzed surface (Figure 4a). The resulting defects are a loss of material of the working surface of the punch. During contact under pressure of two surfaces, abrasive wear occurs most often. It can be carried out in two ways: the first is the contact of three bodies, and the second is the contact of two bodies. In both of these cases, there is a micro-scale abrasion with harder and less harder material particles. If the separate particles are very hard and large, then they stick into the material of a lower hardness and may scratch the surface on their way (grooves). The intensification of the course of abrasion and the intensification of other mechanisms of surface degradation will occur in the case of the accumulation of abraded microparticles [10,11].

During the nibbling process, the flank face abrasive wear and fatigue wear were mainly found. The coated stamp was characterized by significantly lower abrasive wear after 70,000 working cycles (Figure 4b). Traces of form fatigue by spalling and pitting were visible. It was concentrated directly on the cutting edge of the punch and was not present on the side surfaces of the punch. In the case of the 120,000 cycles of work around the entire circumference of the punch, the contact with the sheet material was mainly caused by the abrasive wear mechanism (Figure 5a). As a result of the cyclical repetition of this phenomenon, there was a gradual degradation of the surface of the tools with a mixed destruction mechanism: grooving and local chipping. The coated punch had a smaller abrasive wear zone. It was characterized by a more regular surface structure and local fatigue wear (Figure 5b).

#### 3.1.2. Tool Hardness

**Figure 6:**
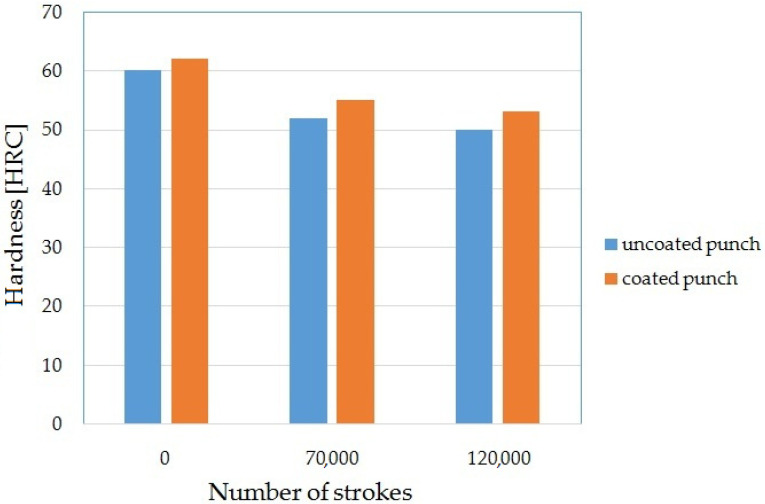
Hardness test results.

**Figure 7:**
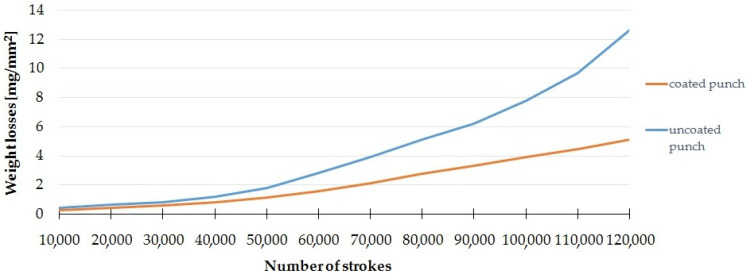
Weight loss and number of strokes.

The results of hardness tests are shown in Figure 6. The results show that the coating increased the hardness of the punch from 60 to 62 HRC. The coating hardness was measured by the Rockwell method. This method leaves a small blemish after the measurement; therefore, the measurement with this method can be used as a non-destructive method during the subsequent stages of research. It was found that the hardness of the coating decreased with increasing cycles. For 70,000 working cycles, the hardness of the punch without coating decreased by 13%. For the coated punch, there was a decrease of 11% for the same number of work cycles. For 120,000 cycles of operation, the hardness decreased by 17% for the uncoated punch and by 15% for the coated punch. The reason for obtaining such results is that the coating rubbed off during operation, thereby changing its thickness. It is difficult to measure the hardness of thin coatings because, in this case, the hardness of the substrate is also involuntarily tested; in this case, the NC11LV steel. Undoubtedly, it is a good comparative method to assess the change in the wear of the AlCrTiN—TIGRAL coating layer, which correlates with the diagram in Figure 7. Cryogenic treatment can also be used to increase the hardness of the stamps. The authors of the work in [4] showed that it is possible to increase the hardness of punches by about 2%

#### 3.1.3. Tool Mass Loss

The wear of the punch can be assessed on the basis of the decrease in its mass, which, in this study, was characterized as the mass loss of wear (mg) per unit area (mm^2^). The area measurements were made with the ATOS Triple Scan system. Samples were tested every 10,000 cycles. The results are the mean of the measurements for five coated and five uncoated punches.

Figure 7 shows the dependence of the loss of mass on the number of working cycles for a punch with and without a coating. The largest increase in weight loss was obtained after exceeding 50,000 work cycles. A significant increase in the impact of the coating on limiting this phenomenon for subsequent work cycles can be noticed. After 100,000 cycles, the weight loss for the punch with the coating was about 4 mg/mm^2^ less than without the coating, whereas, after 120,000 cycles, the weight loss for the punch with the coating was about 7 mg/mm^2^ less than without the coating. Due to the fact that, in the analyzed case, no significant adhesive wear (build-up) was observed, it was assumed that the weight difference only results from abrasive wear.

### 3.2. The Results of the Form Fatigue Wear Process

#### 3.2.1. Analytical Solution

**Figure 8:**
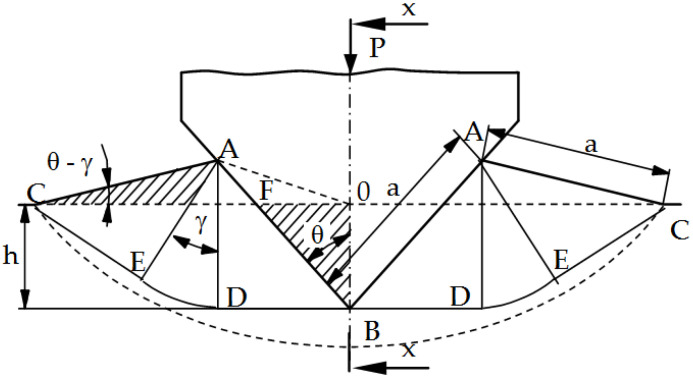
Scheme of deformation during insertion of wedge into plastic material [26].

In order to assess the influence of the tool geometry on the intensity of the form fatigue wear process (spalling and pitting), numerical studies of the punching process were carried out. The intensity of spalling and pitting is closely related to the pressures in the contact zone. By analyzing the pressures in the contact zone (Hertz pressures) and the state of stresses and deformations in the tools, it is possible to predict the durability (resistance to fatigue wear) by determining, for example, the Wöhler curve [24]. On the basis of the literature [25,26], in the processes of working and plastic working (plastic cutting), the maximum coefficient of static friction is µ = 0.39. Exceeding this value results in the shearing of the material under the surface and its peeling. It was also found that the first stage to explain the fatigue wear mechanism is the assessment of the value and distribution of pressures in the contact zone when pressing a rigid wedge with an apex angle of 2θ into the ideally rigid plastic medium (the so-called Saint-Venant body) (Figure 8). For such a body, the material hardening coefficient E_T_ = 0. This problem for plane states was solved by R. Hill [25] using the characterization method (material slip lines, the so-called Luders lines) and is included in the works of W. Szczepiński [26]. The presented solution can be used in industrial practice only for rectilinear cutting edges and materials that do not show hardening. At the same time, this simplified solution is a good basis for estimating the value of average pressures; thus, it provides guidelines for the design of tools and estimating their durability (in particular, the selection of θ angles).

Solving the boundary problem for stresses, the formula for the evenly distributed wedge pressure p on the material along the contact segment AB was obtained [25,26]:

p = 2k (1 + γ),(1)

where k is the shear yield strength and, for the nonlinear Huber–Mises–Hencky yield condition, k = R_e_/$\sqrt{3};$ R_e_ is the yield point of the material (Figure 8); γ is the angle of the polar segment EAD [25,26].

**Figure 9:**
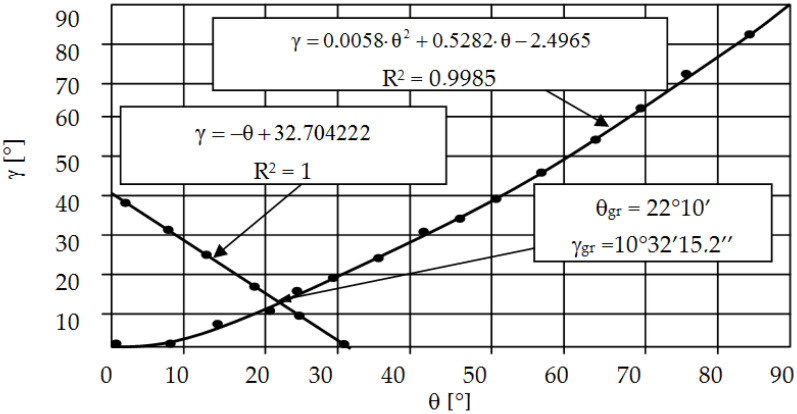
Graphs γ=f(θ) and assignment of border angle θ_gr_.

**Figure 10:**
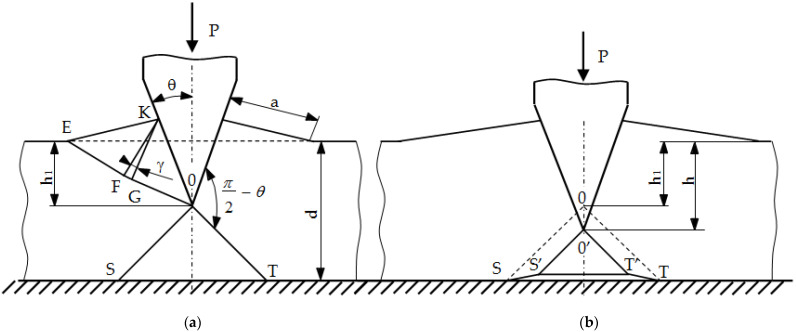
Cutting by wedge-shaped tool: (**a**) phase I; (**b**) phase II.

The process of cutting a block made of a plastic material of thickness d with the apex angle 2θ under the action of the P force is considered (Figure 9). No friction between the contact surfaces is assumed: tool (wedge)–object, object–substrate [26,27,28,29,30]. The GOS area (Figure 10a) may undergo rigid displacements (first case) or undergo plastic deformations (second case). The value of the critical angle θ_gr_, which separates these two cases, was derived from the nonlinear Huber–Mises–Hencky plasticity condition. The following formula was obtained:

(2) $$\theta_{\mathrm{gr}}=\frac{\pi }{2}-1-\gamma$$

From the condition of maintaining a constant field (incompressible material), the following relationship is obtained:

(3) $$a^{2}\left[\cos\left(\theta -\gamma \right)+\sin\theta \right]\cdot \sin\left(\theta -\gamma \right)=a\cdot h\cdot \sin\theta ,$$

where a is the length of the contact area, calculated from the formula:

(4) $$a=\frac{h}{\cos\theta -\sin\left(\theta -\gamma \right)},$$

and γ is the angle between the sections KF and KG (Figure 10a).

Based on Dependence (2) and (3), after making the transformations, the following formula was obtained:

(5) $$\cos\left(2\theta -\gamma \right)=\mathrm{tg}\left(\frac{\pi }{4}-\frac{\gamma }{2}\right),$$

The values of the limit angles θ_gr_ and γ_gr_ are calculated by solving the system of Equations (1) and (5), assuming: θ = θ_gr_, γ = γ_gr_. Its entangled form means that an approximate solution is necessary, e.g., iteratively or graphically. In this study, the values of these angles: θ_gr_ = 22°10′ and γ_gr_ = 10°32′15.2″ were determined graphically (Figure 9). The presented proprietary solution makes it possible to determine the γ angle depending on the θ angle used on the tool, and, thus, to evaluate the average contact pressures.

The entire process was divided into two phases (Figure 10).

Phase I

Phase I (Figure 10a) lasts until the indentation h of the wedge under the action of the force P on the object reaches such a critical value h_1_ that it causes its splitting. The then occurring critical value of the depression is expressed by the formula:

(6) $$h_{1}=\frac{d\left[\cos\theta -\sin\left(\theta -\gamma \right)\right]}{1+\gamma +\cos\theta -\sin\left(\theta -\gamma \right)}$$

where: d is the thickness of the workpiece to be cut.

Phase II

During the further process of engaging the rigid wedge (Figure 10b), the material of the workpiece under the tool turns into a plastic state. The material shifts to the sides. The spacing value for a wedge cavity to a depth h > h_1_ is (h − h_1_)·tgθ on each side. The same value of the gap exists between the momentary deformation area O’S’T’ under the wedge and the ground. The end of the cut process occurs when S’ and T’ meet, i.e., when h = h_2_, where:

(7) $$h_{2}=\frac{h_{1}\cdot \mathrm{tg}\theta +d}{1+\mathrm{tg}\theta }$$

The proposed analytical solution (especially the graphic proprietary solution of dependencies γ = f (θ)) enables a quick estimation of the value of mean pressures in the contact zone of the tool with the object in the process of plastic cutting. This is a good solution for cutting materials that do not strengthen and have a pro-linear cut edge. In the case of cutting strengthening materials, the value and distribution of pressures is significantly different from the analytical solution. The pressure exerted in the contact zone leads to the formation of a stress accumulation point (Bielajew point) under the surface of the material being cut and in the surface layer of the tool. In the case of the material being cut, it will lead to a brittle fracture, whereas, in the case of tools (punches) it will lead to fatigue wear (pitting, spalling). Too high a pressure value or an improperly distributed pressure will cause excessive stresses at the Bielajew point, causing a local loss of cohesion of the material (microcrack), which is the initiation of fatigue wear.

In order to more accurately determine the value and distribution of contact pressures, physical, mathematical and computer modeling was used. In this approach, it is also possible to estimate the material fatigue phenomenon, i.e., to determine the durability and the Wöhler curve to forecast the operational properties of nibbling tools.

#### 3.2.2. Numerical Solution

**Figure 11:**
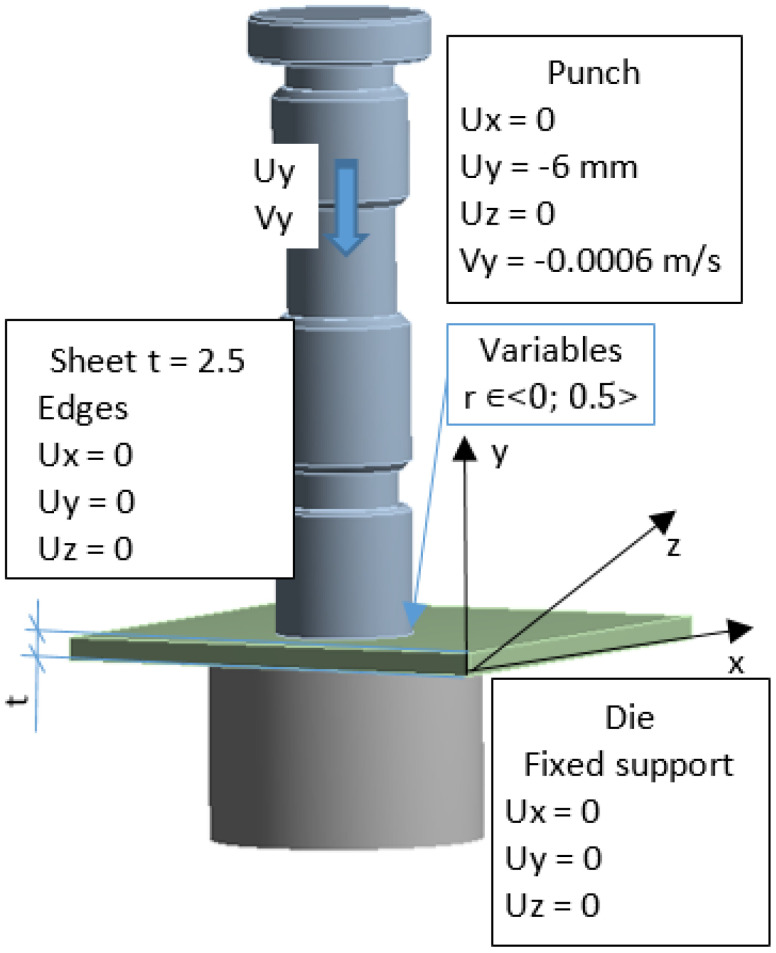
The initial and boundary conditions in modeling of nibbling process.

The first stage of numerical modeling is the development of the physical and mathematical model of the nibbling process. A list of phenomena, assumptions and simplifications was separated, and a solid model of tools (die and punch) and an object punched out of sheet metal with a thickness of t = 2.5 mm was developed (Figure 11). The nibbling process can be simplified to a single punching process, provided that the bending moments of the tool (punch) do not change the distribution and pressure values in the contact zone and thus do not cause a deflection of the side punch (small deflection arrow). This simplification is possible only for the case of using large tool diameters and small sheet thicknesses. In the analyzed case, such a simplification was experimentally verified, and a slight deflection of the tool resulting from the bending process was confirmed. In addition, in the nibbling process (punching only with part of the punch), the tool rotates, leading to an even wear of the cutting edge around its entire circumference. For the geometric model (Figure 3), the following initial boundary conditions were developed (Figure 11). The elastic-visco-plastic material model was used to describe the properties of the sheet metal.

In the geometry model, the geometry of the tools was modified—the radius of the rounding of the contact areas r ∊ <0; 0.5 > mm. It was found that the analytical solution makes it possible to assess contact pressures only with far-reaching simplification. The technique does not use perfectly sharp cutting edges (no technological possibilities), and such solutions are of a low durability. However, fillet radii may be used, which improve the tool life while reducing the quality of the finished product. The radius was modified in order to assess its influence on the achieved tool life.

**Figure 12:**
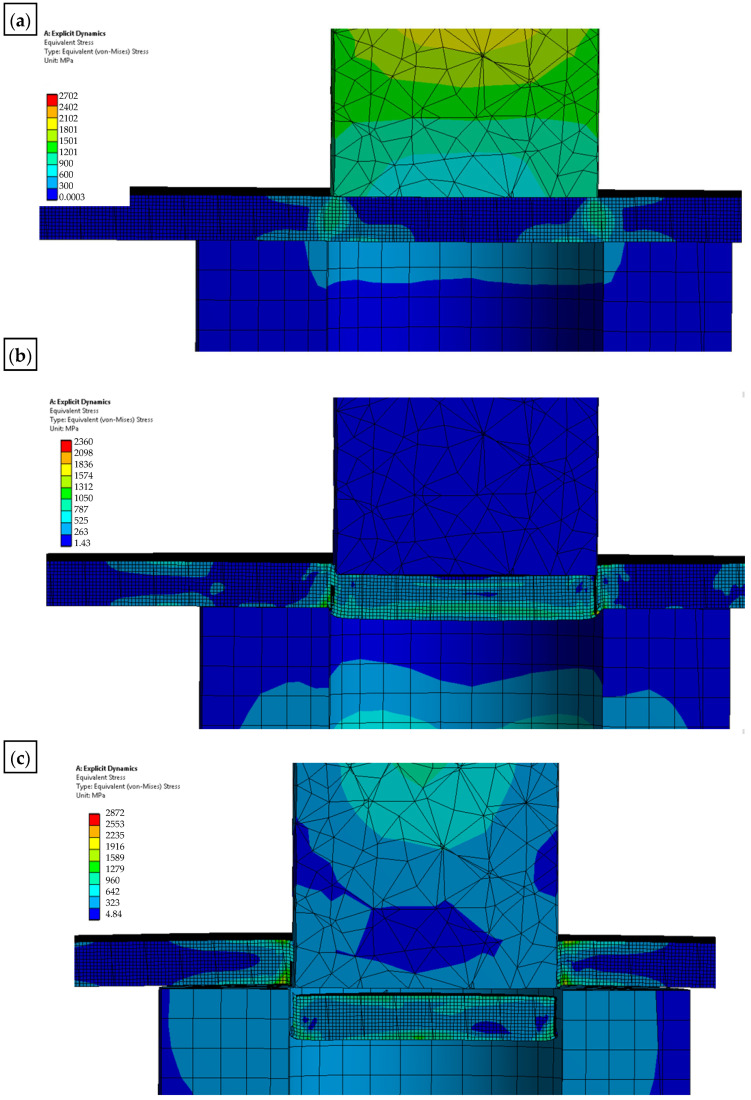
The state of reduced stresses in the tool and the punched sheet for various levels of advancement of the process: (**a**) for 3% advancement; (**b**) for 10% advancement; (**c**) for 80% advancement [MPa].

**Figure 13:**
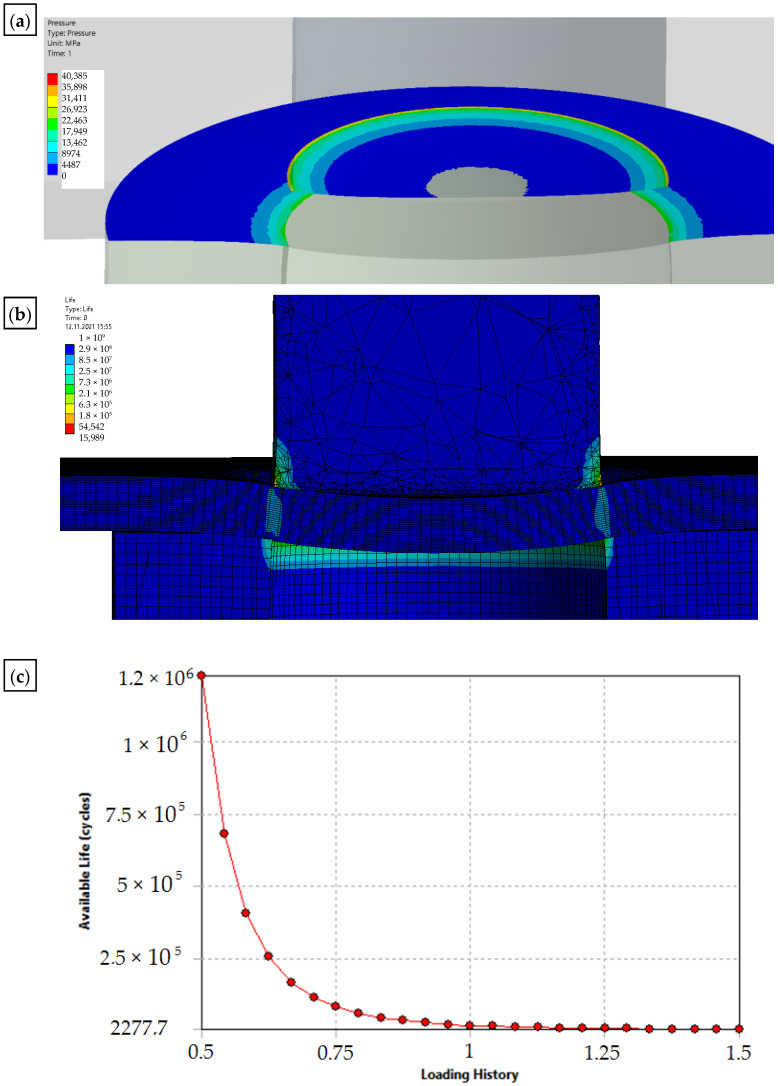
Results of the simulation of the punching process with the use of a sharp punch (without rounding of the cutting edge—r = 0: (**a**) state of pressure in the contact zone [MPa]; (**b**) durability; (**c**) fatigue sensitivity.

**Figure 14:**
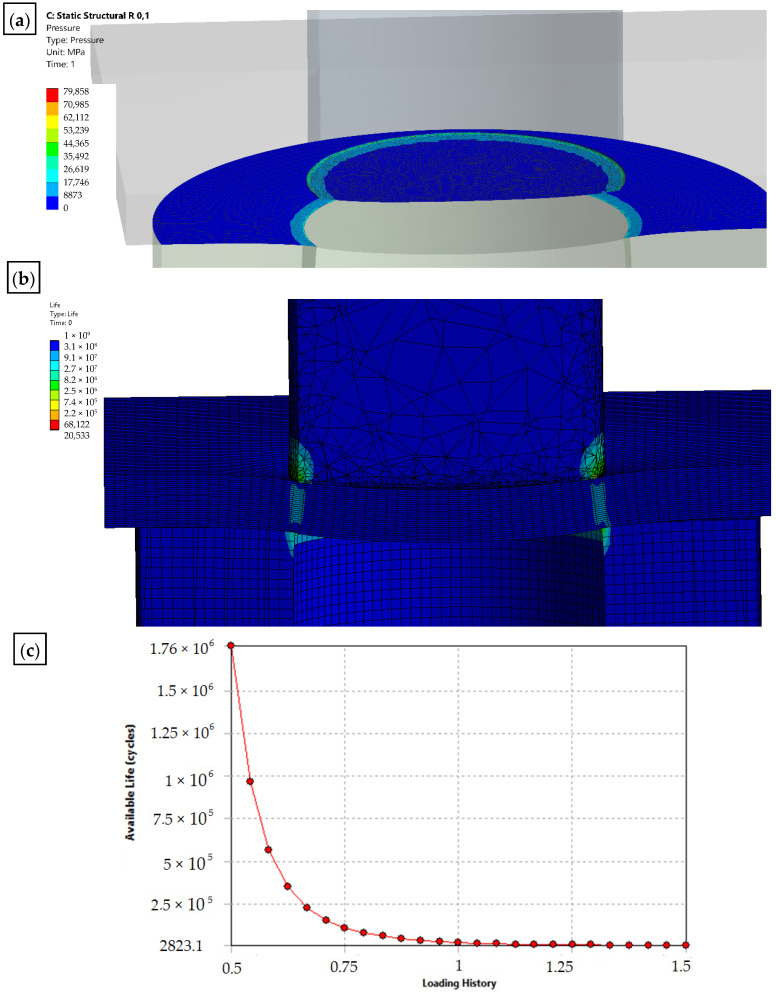
Results of the simulation of the punching process with the use of a punch with rounding the cutting edge—r = 0.1: (**a**) state of pressure in the contact zone [MPa]; (**b**) durability; (**c**) fatigue sensitivity.

The solid model was discretized with finite solid elements. The boundary initial conditions simulating the process of a single punching were superimposed on the developed discrete model. The developed equation of the motion of the object was solved with the use of the explicit integration method [29,30,31,32]. The simulation results are shown in Figure 12, Figure 13 and Figure 14. The nibbling process consists of separating the material with the use of a tool that causes pressure on the shaped material, creating an appropriate state of deformation in the cross-section of the sheet and the cutting zone. Numerical results show that the greatest stresses and pressures in the contact zone of the tools (die and punch) occur in the initial phase of the process (until the first loss of material cohesion). During further penetration of the punch, the value of the pressures does not increase. It has been found that higher pressures and, thus, a lower fatigue wear resistance, are related to the punch. The greatest pressures and stresses occur at the edge of the punch.

A modification of the contact area (rounding of the cutting edge) of the punch with the sheet will result in the value and distribution of the pressure on the punch being more favorable in terms of its operation (the tool life will increase), while deteriorating the technological quality of the product (a larger burr will be created and the separation surface will be more rough). The developed numerical models enable the simulation of the process for selected radii of rounding the cutting edge of the punch. The results for r = 0 and r = 0.1 mm are presented below (Figure 13 and Figure 14).

**Figure 15:**
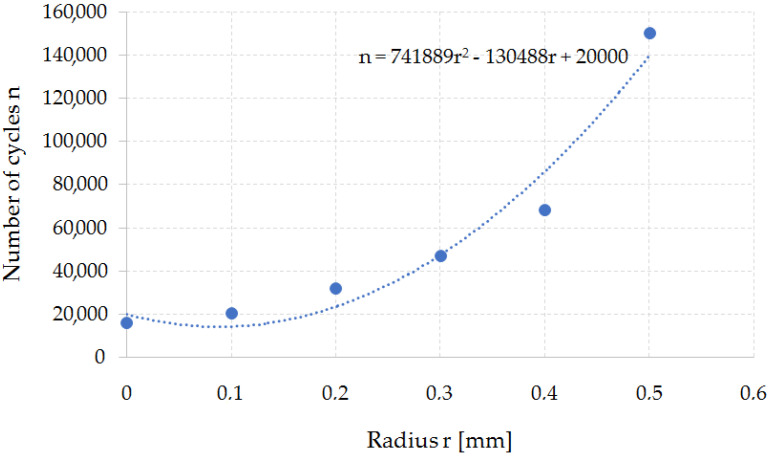
Graph of the dependence of the fatigue wear resistance as a function of the punch cutting edge rounding radius n = f (r).

On the basis of the simulations, a dependence curve of changes in the fatigue wear resistance as a function of the punch cutting edge radius was developed (Figure 15).

The developed function makes it possible to estimate the tool’s resistance to fatigue depending on the geometrical parameters of the tool. It is the necessary knowledge for the correct design of tools and the technological process of the punching process and, as a result, the nibbling process. Changing the punch cutting edge radius has a significant impact on the quality of the finished product. On the basis of the developed function, the technologist can select the tool geometry (rounding radius) depending on the tool life that they want to achieve. At the same time, it should be noted that increasing the radius of the fillet leads to an increase in the fatigue wear resistance, while deteriorating the quality of the finished product (increasing the roughness of the separation surface). Therefore, further work in this area will require process optimization due to the durability of the tools and the quality of the finished product. As a rule, increasing the rounding radius leads to a deterioration of the product quality (excessive burrs and surface deflections appear—surface flatness error) and an increase in shaping forces. The issue of the influence of the cutting tool geometry on the quality of the finished product is discussed below.

### 3.3. Quality of Workpiece

#### 3.3.1. Quality of Workpiece Cut Surface

**Figure 16:**
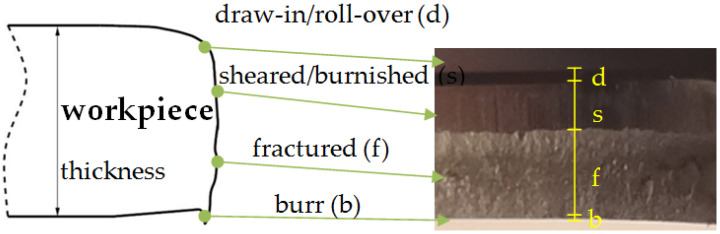
Zones on workpiece cut surface.

The quality of the product in the nibbling process is determined on the basis of the width of individual zones on the cut surface (Figure 16) and the deviation of the diameter of the obtained holes.

**Figure 17:**
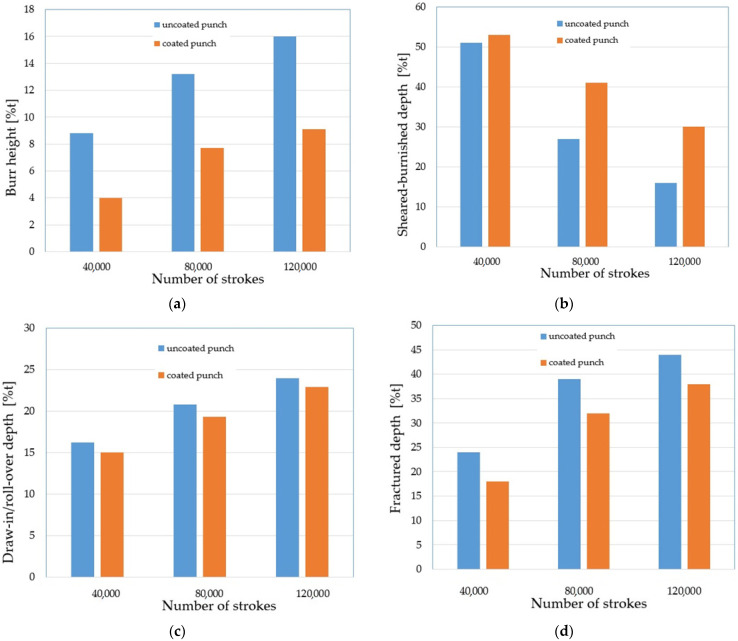
Average values of the cut surface zones for uncoated and coated punches: (**a**) burr height; (**b**) sheared–burnished; (**c**) draw-in/roll-over; (**d**) fractured.

The SEM images of the cut edge of the holes were used in this study to evaluate the part geometry by examining the burr height, sheared–burnished zone, draw-in/roll-over and the fracture on the dependence of the tool wear. Measurements were made at 20 places around the perimeter of the holes. The results were averaged for each zone. The results are shown in Figure 17, where %t is the percentage of the zone width in relation to the thickness of the sheet t (100%t = 2.5 mm). The authors’ previous research shows that one of the key problems in shaping products using mechanical cutting techniques is the formation of burrs on the cut surface [26,32]. The height of burrs can be reduced by an appropriate selection of the technological parameters of the process; mainly the cutting clearance depending on the thickness of the material and its type. The research carried out in this study also showed a significant influence of the degree of tool wear on the burr height. The increase in the degree of wear resulted in an increase in burrs. Burr heights are reduced when a coated punch is used. Even for a small number of cycles of operation, a significant increase in burrs for the punch without a coating can be noticed (Figure 17a). For 120,000 working cycles, the height of the burrs on the cut edge is twice as high for the uncoated punch than for the coated punch. As the degree of wear increases, the width of the sheared–burnished zone (Figure 17b) decreases, whereas the width of the fractured zone (Figure 17c) increases. The width of the sheared–burnished zone for 40,000 cycles was similar for each of the punches (Figure 17b). A significant drop occurred at 80,000 cycles. For the punch with the coating, the decrease was about 12% t; for the uncoated punch, it was about 24% t. For 120,000 cycles, the width of the sheared–burnished zone decreased by 23% t for the coated punch and by 35% t for the uncoated punch. For the same number of cycles, the width of the fractured zone was 38% t for the coated punch and 44% t for the uncoated punch. The draw-in/roll-over width depends mainly on the value of the cutting clearance and increases as the clearance increases. As the research carried out has shown, the extent of the wear also has an impact on the width of this zone, although it is smaller than in the case of the burr and the smooth zone. Compared to 40,000 cycles at 120,000 cycles, an increase in the draw-in/roll-over of about 8% t was obtained for the two punch variants. For the stamp with the coating, the width of this zone was about 2% smaller than for the stamp without the coating (Figure 17d).

#### 3.3.2. The Hole Geometry

**Figure 18:**
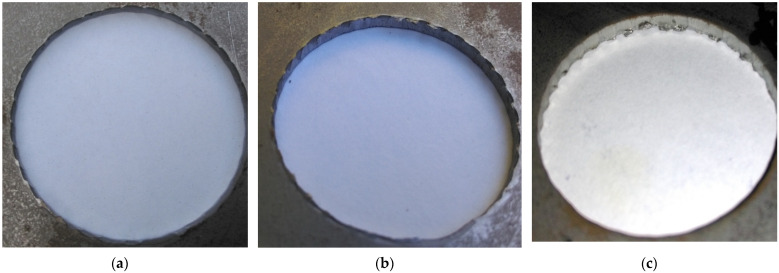
View of sample holes after the process: (**a**) 40,000 cycles; (**b**) 80,000 cycles; (**c**) 120,000 cycles.

**Figure 19:**
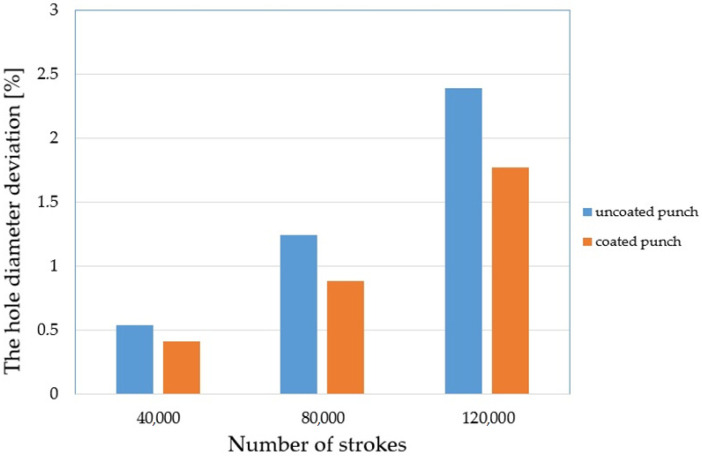
The hole diameter deviation.

Tool wear impairs the geometry of the punch and a geometrically deteriorated punch creates high tribological loads in the sheet metal punching process. Therefore, the hole geometry is negatively affected [4,27,29]. Figure 18 shows the appearance of the obtained holes after selected work cycles. As the cycle of work increases, the formation of areas on the perimeter of the holes with built-up zones and burrs can be observed (Figure 18c). The conducted research is in line with the trend found in literature. The hole diameter deviation increases (Figure 18b,c) due to the change in the geometry of the cutting edge of the punch. By spreading the coating over the tool, smaller deviations can be achieved. The conducted tests showed a deviation of 0.41% for a stamp with a coating and 0.53% for a stamp without a coating for 40,000 work cycles (Figure 19). For 120,000 cycles, the difference between the punches was 0.62%. The values of the deviation were 1.77% for the stamp with the coating and 2.39% for the stamp without the coating (Figure 19).

## 4. Conclusions

The implementation of theoretical analyses and numerical and experimental research allows for the development of the following conclusions:

− During the nibbling process, the following types of wear may occur on the punch edge: abrasive wear, form fatigue and adhesive wear;
− In the analytical solution, formulas for determining the pressures in the contact zone were developed, thus facilitating the design of nibbling tools;
− The process of abrasive wear was primarily investigated in the experimental research. For this purpose, the nibbling process was tested on S235JR + AR steel sheets with tools made of NC11LV/1.2379 steel without any coating and with an AlCrTiN layer covering. It was found that the special AlCrTiN layer used enables an increase in the abrasive wear resistance, and thus an increase in durability by approx. three times. At the same time, it should be noted that the increase in the abrasive wear resistance (high coating hardness) may have a negative impact on fatigue wear (the surface layer of the tools may peel off). Therefore, in order to properly design the technological process (the quality of the finished product and durability of the tools), the process should be poly-optimized in accordance with the adopted criteria;
− The influence of the punch rounding radius on the fatigue wear was investigated in the numerical studies. It has been shown that the change in the punch cutting edge radius from r = 0 mm to r = 0.5 mm enables an approx. seven-fold increase in the fatigue wear resistance. It was found that the change in the punch cutting edge radius rounding off influences the quality of the product (the larger the radius r, the worse the technological quality of the product);
− The last element of the work is the assessment of the technological quality of the product after nibbling depending on the degree and type of stamp wear (quantitative and qualitative assessment). It has been shown that used nibbling punches cause a deterioration of the technological quality of the finished product. The tests were carried out by measuring the diameters of the nibbled holes. It was found that after 120,000 nibbling cycles, the diameter deviation amounts to approx. 1.8% for punches covered with the AlCrTiN layer and 2.4% for punches without the special coating.

## Figures and Tables

**Table 1:** Mechanical properties of NC11LV steel.

Density (kg/m^3^)	Yield *R*_p0.2_ (MPa)	Tensile *R*_m_ (MPa)	Impact Strength for U-Notch Samples in Charpy Impact Test KU/S (J/cm^2^)	Elongation *A* (%)	Reduction in Cross Section on Fracture *Z* (%)
7650	534	963	14	7	10

**Table 2:** Chemical composition of NC11LV steel (%).

C	Si	Mn	Cr	Mo	Ni	V
1.5	0.15	0.15	11	0.7	0	0.9

**Table 3:** Mechanical properties of S235JR + AR steel.

Density (kg/m^3^)		Yield Stress *R*_p0.2_ (MPa)	Tensile *R*_m_ (MPa)	Elongation *A* (%)	Shear Module (GPa)
7837		315	426	25	80
Standard deviation	23.6	20.4	19.7	2.2	2.6

**Table 4:** Chemical composition of S235JR + AR steel (%).

C	Mn	P	S	N	Cu
0.162	0.33	0.012	0.009	0.0019	0.054

**Table 5:** AlCrTiN—TIGRAL coat characteristic properties.

Hardness HV	Maximum Application Temperature [°C]	Coefficient of Friction against Steel [-]	Layer Thickness [µm]
3300 ± 300	900	0.6	3–5

## Data Availability

Data sharing is not applicable to this article.
